# Recurrence risk is associated with preoperatively advanced prolapse stage: Is there a difference between women with stage 2 and those with stage 3 or 4 cystocele?

**DOI:** 10.1007/s00192-016-3216-0

**Published:** 2016-12-06

**Authors:** Tineke F. M. Vergeldt, Kim J. B. Notten, Kirsten B. Kluivers, Mirjam Weemhoff

**Affiliations:** 1 Obstetrics & Gynecology, Radboud University Medical Center, P.O.Box 9101, 6500 HB Nijmegen, The Netherlands; 2 Obstetrics & Gynecology, Maastricht University Medical Center, Maastricht, The Netherlands; 3 Obstetrics & Gynecology, Zuyderland Medical Center, Heerlen, The Netherlands

**Keywords:** Advanced stage, Age, Pelvic organ prolapse, Recurrence

## Abstract

**Introduction and hypothesis:**

Pelvic organ prolapse (POP) recurrence after surgery is a major problem. POP that is more advanced preoperatively is associated with a higher risk of recurrence postoperatively. We hypothesized that women with a stage 2 cystocele differ from those with a stage 3 or 4 cystocele. The aim of this study was to compare the baseline characteristics of women with mild and those with more advanced cystocele.

**Methods:**

Patients had participated in one of two multicenter prospective cohort studies on women undergoing conventional anterior colporrhaphy without previous POP surgery. This was a secondary analysis of these data. Women with a preoperative cystocele stage 2 were compared with women with a stage 3 or 4 cystocele. Logistic regression models were employed to calculate odds ratios (OR) and 95% confidence intervals (CI).

**Results:**

Two hundred and sixty-nine women were assessed, of whom 132 (49.1%) had an advanced cystocele. Only older age was significantly associated with advanced cystocele preoperatively, with an OR of 1.07 (95% CI 1.04–1.10). There were no significant differences between women with advanced or stage 2 cystocele in body mass index, vaginal deliveries, assisted delivery, positive family history of POP, concurrent rectocele, concurrent uterine of vaginal vault prolapse, major levator ani muscle defects, or levator hiatal area.

**Conclusions:**

Women with advanced cystocele were significantly older than women with stage 2 cystocele. This raises the question whether it would be favorable to perform POP surgery in an earlier stage, i.e., at a younger age, in order to prevent POP recurrence.

## Introduction

Female pelvic organ prolapse (POP) is a common condition that can have a great negative impact on women’s social, physical, and psychological well-being [1]. Women may have symptoms such as feeling or seeing a bulge in their vagina, or they may have micturition, defecation, and sexual problems. The severity of symptoms does not correlate well with the severity of POP, and many women with POP are asymptomatic [2]. In case of symptomatic POP, options include expectant management (pelvic floor exercises, including physical therapy), pessary treatment, and surgery [3]. The lifetime risk of surgery for POP in the general female population is 13–19% [4, 5]. POP recurrence after surgery is a major problem, with anatomical recurrence rates reported in the literature from 31% up to 59% [6, 7]. Anterior-compartment prolapse, also referred to as cystocele, is the most commonly affected in POP and is the most prone for recurrence after surgery [8, 9].

Advanced preoperative stage seems to be an important risk factor for recurrence [7, 10–13]. In a systematic review on risk factors, it was the only risk factor associated with recurrence in at least two cohort or cross-sectional studies with multivariate analysis in a Western country [14]. This raises the question of whether there is a difference between women with mild and women with more advanced POP.

We hypothesized that women with a stage 2 cystocele differ from women with a stage 3 or 4 cystocele. The aim of this study was to compare baseline characteristics of women with mild to women with more advanced cystocele.

## Materials and methods

Our cohort had participated in one of two multicenter prospective cohort studies concerning women undergoing conventional anterior colporrhaphy (i.e., without the use of mesh materials) performed in nine teaching hospitals in The Netherlands. This was a secondary analysis of these data. In the first study, women were assessed between January 2006 and September 2008 for a randomized controlled trial comparing indwelling catheterization for 2 versus 5 days following surgery [15]. The follow-up visits after 2 years, which included POP staging and translabial 3D ultrasound (US), were performed between November 2009 and April 2010. The aim of that study was to determine whether levator defects were a risk factor for cystocele recurrence and to identify other risk factors associated with recurrence [12]. In the second study, women were recruited from June 2010 until November 2012, with a follow-up of 1 year. The primary aim of this study was to assess the diagnostic accuracy of translabial 3D US in the diagnosis of levator defects in women with POP, using magnetic resonance imaging (MRI) as reference test [16, 17]. The protocols of both studies were approved by the medical ethics committees of each participating hospital, and all women gave written informed consent before enrollment in the study. In both studies, women had undergone an anterior colporrhaphy without the use of mesh materials and without previous POP surgery. Anterior colporrhaphy was performed alone or in combination with other POP procedures.

All women completed a validated questionnaire, with additional questions concerning possible risk factors [18]. Pelvic examination was performed with the patient in the lithotomy position. POP staging was performed according to the Pelvic Organ Prolapse Quantification (POP-Q) scoring system in the second database [19] and according to the Baden-Walker classification in the first [20], as POP-Q classification was not generally introduced into daily practice at the time of that study. For this secondary analysis, only women with Baden-Walker or POP-Q stage 2 cystocele were included. Advanced preoperative stage was defined as Baden-Walker or POP-Q stage 3 or 4, i.e., the most distal part of the prolapse during Valsalva was bulging out of the vagina according to the Baden-Walker classification or ≥1 cm from the hymenal remnants according to the POP-Q classification. The procedure of translabial 3D US and assessment of US finding is described previously [13].

Women undergoing an anterior colporrhaphy for a stage 2 cystocele were compared with women undergoing the same procedure for a stage 3 or 4 (advanced) cystocele. Baseline characteristics investigated were age, body mass index (BMI), number of vaginal deliveries, having had an assisted vaginal delivery, positive family history of POP, concurrent rectocele, concurrent uterine or vaginal-vault prolapse, major levator ani muscle defects on US, and levator hiatal area during Valsalva on US. Positive family history of POP was defined as having (had) a mother or sister with POP. Because two databases were combined, baseline characteristics were calculated for both databases separately and combined.

We used the statistical software package SPSS version 22.0 (SPSS Inc., Chicago, IL, USA) for statistical analyses. Logistic regression models were employed to calculate odds ratios (OR) and 95% confidence intervals (CI). A *p* value < 0.05 was considered statistically significant.

## Results

In this secondary analysis, 269 women with at least Baden-Walker or POP-Q stage 2 cystocele were included: 157 (50.9%) underwent anterior colporrhaphy for stage 2 cystocele and 132 (49.1%) for stage 3 or 4. Baseline characteristics for the two databases separately and combined are shown in Table 1. There were no significant differences except for concurrent uterine of vaginal vault prolapse, which was more frequent in the first database.

**Table 1:** Patient baseline characteristics

	Total (*n* = 269)		Weemhoff [12, 15] (*n* = 130)		Notten [16, 17] (*n* = 139)		*P* value
	*n*	*n* or mean (% or range)	*n*	*n* or mean (% or range)	*n*	*n* or mean (% or range)	
Preoperative stage 3 or 4 (n)	269	132 (49.1%)	130	70 (53.8%)	139	62 (44.6%)	0.13
Age (years)	269	58.1 (31–87)	130	59.0 (39–87)	139	57.4 (31–78)	0.20
BMI (kg/m^2^)	241	26.0 (17.5–41.9)	125	26.2 (18.4–36.4)	116	25.7 (17.5–41.9)	0.26
Vaginal delivery (mean)	258	2.3 (0–9)	130	2.3 (0–9)	128	2.3 (1–7)	0.65
Assisted delivery (n)	258	31 (12.0%)	130	18 (13.8%)	128	13 (10.2%)	0.36
Family history of POP (n)	252	109 (43.3%)	130	53 (40.8%	122	56 (45.9%)	0.41
Concurrent rectocele (n)	269	115 (42.8%)	130	58 (44.6%)	139	57 (41.0%)	0.55
Concurrent uterine or vaginal vault prolapse (n)	269	161 (59.9%)	130	88 (67.7%)	139	73 (52.5%)	0.01
Major levator defect (n)	262	119 (45.4%)	127	52 (40.9%)	135	67 (49.6%)	0.16
Hiatus during Valsalva (cm^2^)	256	33.3 (16.8–67.4)	122	32.4 (18.4–56.4)	134	34.0 (16.8–67.4)	0.10

*BMI* body mass index, *POP* pelvic organ prolapse

Possible risk factors for advanced cystocele with ORs and 95% CI are shown in Table 2. Only age was significantly associated with advanced cystocele. There were no significant differences between women with advanced or with stage 2 cystocele in BMI, number of vaginal deliveries, having undergone an assisted delivery, positive family history of POP, concurrent rectocele, concurrent uterine of vaginal vault prolapse, major levator ani muscle defects, or levator hiatal area during Valsalva. Because univariate analysis revealed only one significant risk factor, multivariate analysis was not contributory.

**Table 2:** Logistic regression analysis

	Stage 2 cystocele		Stage 3 or 4 cystocele		OR (95% CI)	*P* -value
	*n*	*n* or Mean (% or range)	*n*	*n* or Mean (% or range)		
Age (years)	137	55.1 (31–83)	132	61.3 (39–87)	1.07 (1.04–1.10)	<0.01
BMI (kg/m^2^)	119	26.0 (18.4–41.9)	122	26.0 (17.5–35.2)	1.00 (0.93–1.07)	1.00
Vaginal delivery (n)	129	2.3 (0–4)	129	2.3 (0–9)	1.05 (0.83–1.34)	0.67
Assisted delivery (yes/no),* n* (%)	129	15 (11.6%)	129	16 (12.4%)	1.08 (0.51–2.28)	0.85
Family history of POP (yes/no),* n* (%)	128	51 (39.8%)	124	58 (46.8%)	1.33 (0.81–2.19)	0.27
Concurrent rectocele (yes/no),* n* (%)	136	63 (46.3%)	132	52 (39.4%)	0.76 (0.47–1.24)	0.28
Concurrent uterine of vaginal vault prolapse (yes/no),* n* (%)	137	79 (57.7%)	132	82 (62.1%)	1.20 (0.74–1.96)	0.46
Major levator defect (yes/no),* n* (%)	133	58 (43.6%)	129	61 (47.3%)	1.16 (0.71–1.89)	0.55
Hiatus during Valsalva (cm^2^), mean (range)	130	33.0 (16.8–58.5)	126	33.5 (18.9–67.4)	1.01 (0.98–1.04)	0.59

*OR* odds ratio, *CI* confidence interval, *BMI* body mass index, *POP* pelvic organ prolapse

## Discussion

In this study, characteristics of women seeking surgical treatment for stage 3 or 4 cystocele where compared with those with stage 2 cystocele. Women with advanced cystocele were significantly older.

The knowledge that more advanced POP has a higher risk of recurrence after surgery raises the question as to whether it would be favorable to perform POP surgery at an earlier stage, i.e., at a younger age, rather than to wait until POP has progressed to a more advanced stage. Although older age is a risk factor for primary POP in multiple studies [21–24], other studies have challenged the assumption that POP worsens over time [25–27]. Two cohort studies that investigated the likelihood of POP progression showed high grades of spontaneous regression [25, 26]. Both studies suggested that there was little true change in POP severity over time. A retrospective cohort study to determine the association between patient age and POP has shown a weak and complex relationship [27]. There was a positive correlation between cystocele and age in premenopausal women, but this relationship was reversed after menopause. The authors concluded that ageing appeared to play only a limited role in POP etiology and pathogenesis, contradicting epidemiological studies showing age to be a risk factor for POP surgery [28]. This discrepancy may be due to confounders such as symptoms of bladder or bowel dysfunction that may become more likely with increasing urogenital atrophy, which increases with age. Therefore, surgery might generally be performed at an older age, not because POP has progressed to an advanced stage, but because of more severe symptoms at that age and the assumption that urogenital symptoms are the result of POP when in fact atrophy or overactive bladder syndrome may be the case.

Studies investigating the association between age and POP recurrence show conflicting results, as well [14]. In two studies with a cutoff at 60 years, younger age was a significant risk factor for POP recurrence after surgery [7, 10]. In contrast, in two other studies in which age was a continuous variable and in one study with a cutoff at 70 years, no significant associations were found [11, 12, 29]. The association between age and POP recurrence is thus more complex, with both younger and older age as risk factors for POP recurrence.

Younger women with POP are often advised to postpone POP surgery until progression of complaints because of the high recurrence risk of POP and the risk that multiple surgeries will be needed over time; younger women have a longer life expectancy and therefore more time to develop recurrence. Results of the study reported here argue whether the advice to wait is justified. Theoretically, waiting for progression of complaints could lead to progression of POP due to stretching of tissue and ligaments. On the other hand, stiffening of vaginal tissues after menopause could lead to reduction of distensibility and reduced POP severity. Not every woman with a stage 2 cystocele will develop a stage 3 or 4 cystocele; therefore, performing surgery in every woman with stage 2 cystocele would lead to overtreatment. Possibly, pessary use may reduce the risk of progression to a stage 3 or 4 cystocele due to less tissue and ligament stretching compared with expectant management. In a prospective cohort study, no women experienced POP stage worsening with pessary use [30]. However, the study had a low sample size and short follow-up of 1 year. Among the possible risk factors investigated in the study presented here, only age was significantly associated with advanced cystocele. In future research, it may be interesting to investigate other factors that may be associated with advanced POP, such as biomechanical properties of vaginal tissue or genetic factors.

Strengths of this study are sample size and multicenter design of the studies combined in one database. There were no significant differences between the two databases, except for concurrent uterine or vaginal vault prolapse, which was more frequent in the first database. It is unlikely that this would have influenced our study results, because recurrence rates were comparable between databases. A weakness of the study is the use of two different classification systems for POP staging. Since studies that use either classification system show that advanced POP preoperatively is a risk factor for POP recurrence after surgery, the use of the different systems did not influence our study [7, 10–13]. Because our study population was mainly Caucasian, these findings cannot be extrapolated to other ethnicities.

In conclusion, women with advanced POP have a higher risk of recurrence after POP surgery. Women with advanced cystocele are significantly older than women with stage 2 cystocele. Further research is needed to determine whether it would be favorable to perform POP surgery at an earlier stage (i.e., at a younger age) in order to prevent POP recurrence.
